# Suboccipital Atretic Cephalocele as a Marker for Joubert-Plus Syndrome: An Extended Phenotype of the CPLANE1 Gene Mutation

**DOI:** 10.7759/cureus.99416

**Published:** 2025-12-16

**Authors:** Abdelrahman I Babiker, Haifaa Alkabbani, Sumaya AlMaraghi, Noor Al Sulaiti, Omar Abbas, Ala Aldeen A Al Serhan, Wagdi Al-Kadasi, Magda Yousef, Khalid Mohamed

**Affiliations:** 1 Pediatrics, Sidra Medicine, Doha, QAT; 2 Neurology, Sidra Medicine, Doha, QAT; 3 Neurosurgery, Sidra Medicine, Doha, QAT

**Keywords:** cplane1 gene, joubert syndrome (js), joubert syndrome-related disorders (jsrds), neonatal neurology, occipital encephalocele

## Abstract

Joubert syndrome (JS) is a rare neurological condition characterized by intellectual disability, hypotonia, and an abnormal breathing pattern. MRI brain frequently reveals the presence of the characteristic molar tooth sign. JS is usually inherited in an autosomal recessive manner, although sporadic cases have been reported. JS can present in association with other neurological conditions, such as Dandy-Walker syndrome; this is referred to as Joubert-Plus syndrome. In this report, we will present two patients who presented with a suboccipital swelling following a normal pregnancy and birth by elective Cesarean section at term. Both were found to be normocephalic with no facial dysmorphism and were referred for neurosurgical evaluation. MR imaging demonstrated the presence of Joubert-Plus syndrome. Genetic testing revealed a pathogenic mutation in the CPLANE1 gene. As shown in our two patients, JS can be associated with other central nervous system abnormalities such as the Dandy-Walker malformation in the mesencephalon or caudal fourth ventricle. In patients with atretic encephaloceles, the possibility of an underlying brain malformation or a genetic disorder should always be considered.

## Introduction

Joubert syndrome (JS) is a rare genetic disorder, with an incidence of 1:80,000 - 1:100,000. JS is usually inherited in an autosomal recessive manner, although X-linked and autosomal dominant cases were reported as well as sporadic cases [1]. JS is caused by dysfunction in the main cilium’s structure and function, which in turn results in complicated cerebellar and brain stem malformations. The main manifestation is the absence or underdevelopment of the cerebellar vermis. In addition, a wide range of additional phenotypic abnormalities has been reported [2]. As JS is caused by primary ciliary dysfunction, it can manifest with different degrees of involvement of the neurological, ocular, gastrointestinal, and urogenital systems [3]. The neurological manifestations include intellectual disability, ataxia, abnormal breathing patterns, abnormal eye and tongue movements and low muscle tone (hypotonia). Other abnormalities include facial dysmorphism, polydactyly, low-set ears, microcephaly, speech delay, retinal dystrophies and meningoencephalocele [4]. When Dandy-Walker malformation and other neurological abnormalities coexist with JS, this is referred to as Joubert-Plus syndrome [4]. The following criteria must be present in order to make the diagnosis of JS: hypotonia in infancy, developmental delay or intellectual disability, and the presence of molar tooth sign on MRI [5].

Dandy-Walker syndrome is an uncommon congenital malformation presenting as hypoplasia and upward rotation of the cerebellar vermis and cystic expansion of the fourth ventricle [6]. Ataxia, hydrocephalus, and psychomotor impairment are some of the clinical symptoms associated with the condition. An atretic cephalocele is a congenital intracranial tissue herniation that includes dura, fibrous tissue, and degenerated brain tissue. It can be an isolated finding or present with other syndromes such as Walker-Warburg syndrome, ventriculomegaly, or deformities of the cortical development [7]. To our knowledge, this is the second report of the coexistence of Joubert-Plus syndrome and an atretic cephalocele. In addition, we report on a known genetic mutation associated with oral-facial-digital-syndrome, manifesting as Joubert-Plus syndrome in our cases. 

## Case presentation

Case 1

A four-week-old female infant was born at term as a firstborn to healthy consanguineous parents, by emergency lower segment cesarean section. She was vigorous at birth and did not need NICU admission. She was noted to have a swelling in the back of her head, and cranial ultrasound was requested; the possibility of a Dandy-Walker malformation was raised, and she was evaluated by the neurosurgery team. MRI head was requested. While in the MRI room, she had an episode of desaturation and tachycardia and was admitted for further evaluation; examination revealed a well infant who was normocephalic with no facial dysmorphism or hypotonia with suboccipital swelling. A small umbilical granuloma was noted and treated with silver nitrate. Infection screening was negative. Examination for polydactyly was negative, and there were no clinical signs of oro-facial digital syndrome (OFDS). Full examination and system evaluation, including renal and hepatic ultrasound and detailed eye examination, were requested, and this was concluded as normal.

**Figure 1:**
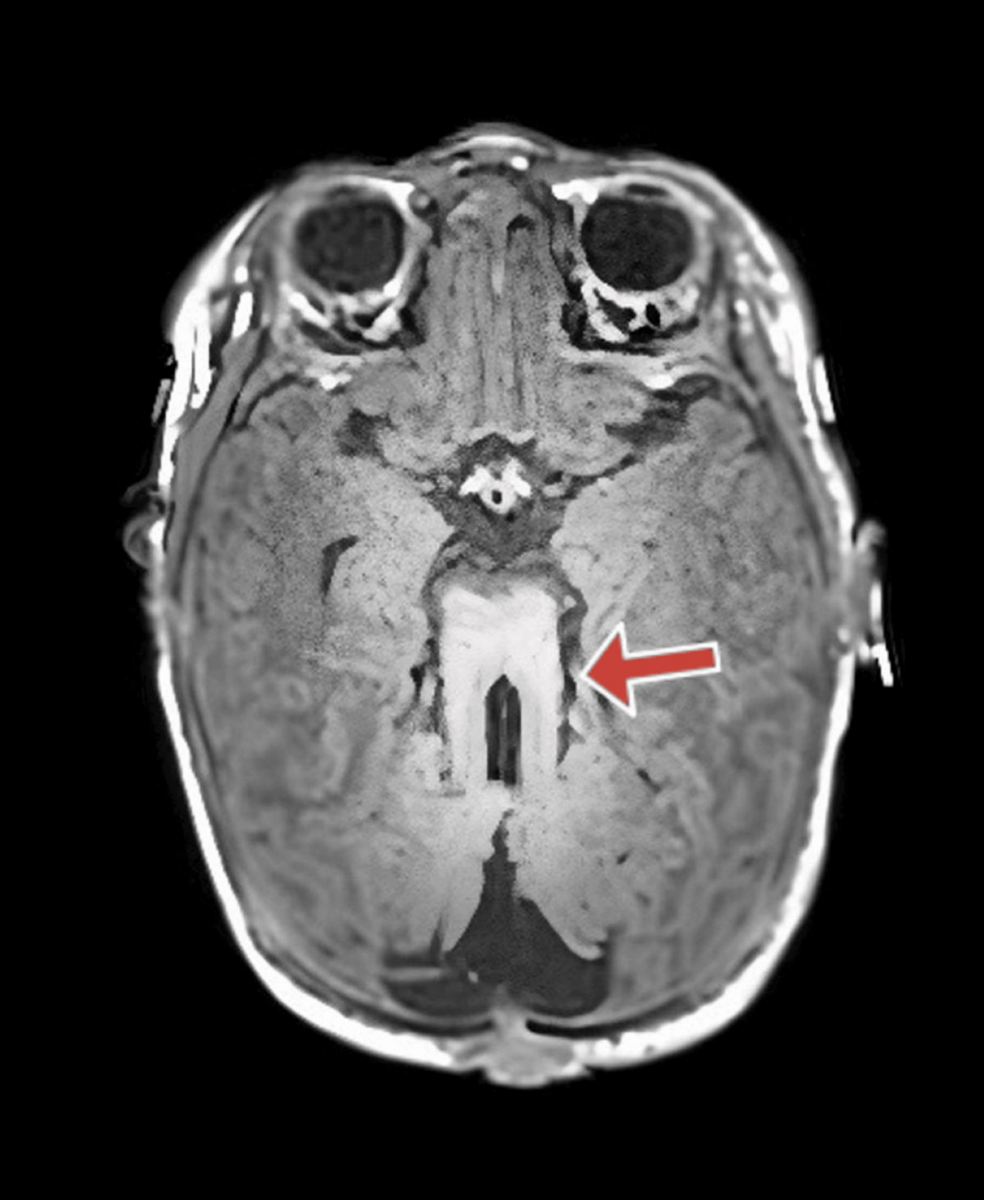
Axial MRI image showing the molar tooth sign characteristic of Joubert syndrome

**Figure 2:**
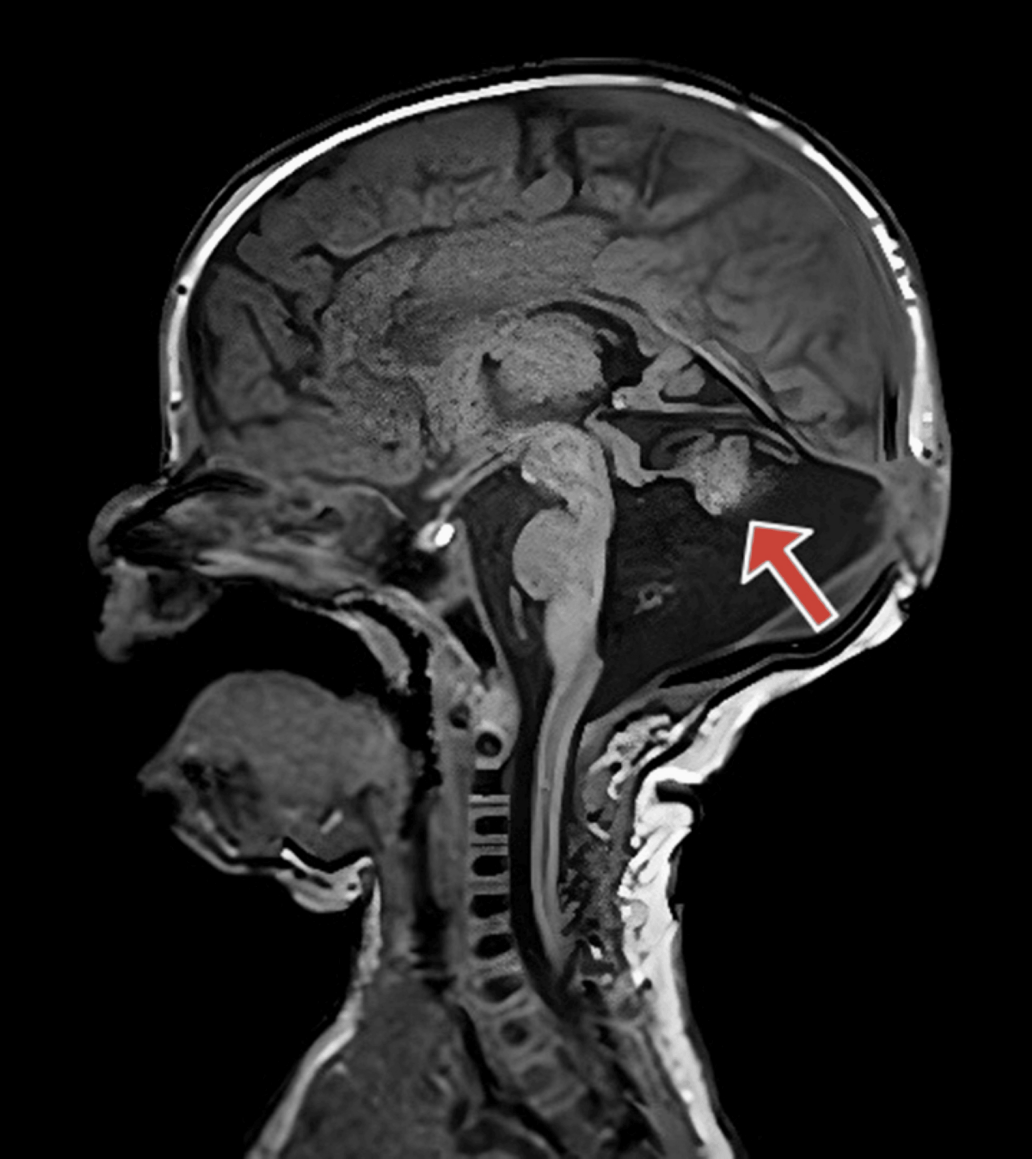
Case 1: MRI brain T1 sagittal image showing the Dandy-Walker feature of cerebellar vermis hypoplasia (arrow) and a small occipital swelling (cephalocele)

The MRI report showed posterior fossa structural dysmorphia with a ‘molar tooth configuration’ of the rostral brain stem and vermo-cerebellar dysplasia (Figure 1). The occipital encephalocele is shown in Figure 2.

The findings mentioned above are keeping with an underlying ciliopathy “Joubert Syndrome and Related Disorders”. The child remained well and did not require any further investigations or medications and was referred to the outpatient clinics for neurosurgery, neurology and clinical genetics. She did not have seizures or any neurological symptoms and had a normal sleep pattern. Examination at six months in the neurology clinic revealed a well child with mild motor delay and hypotonia; she had no seizure, a small swelling and a skull defect were noted, and neurosurgery evaluation was anticipated. The neurosurgery team reviewed her at four months of age and counselled the family about surgery when she is around 12 months of age to close the encephalocele.

Targeted genetic testing revealed a pathogenic homozygous mutation in the CPLANE1 gene confirming JS. 

At the age of 14 months, she remains with mild hypotonia and motor delay, she is able to sit without support and babbles and smiles to her parents. The evolving clinical picture fits the description of mild phenotype of Joubert-Plus syndrome. She does not display features of OFDS. Surgery for the encephalocele is planned and she is enrolled in the neuro-rehabilitation program. 

Case 2

A male infant was born at 36 weeks to consanguineous parents, by emergency lower segment cesarean section due to membrane rupture and an oblique lie. Antenatal ultrasound revealed a structural brain anomaly with an enlarged posterior fossa and a cystic structure, raising concerns for Dandy-Walker Malformation or Meckel-Gruber Syndrome. At birth, the infant appeared vigorous but exhibited swelling at the back of his head with absent bony structures underneath, suggesting an occipital cephalocele. Shortly after birth, he developed desaturation and respiratory distress, leading to NICU admission for respiratory support, where he remained for five months.

**Figure 3:**
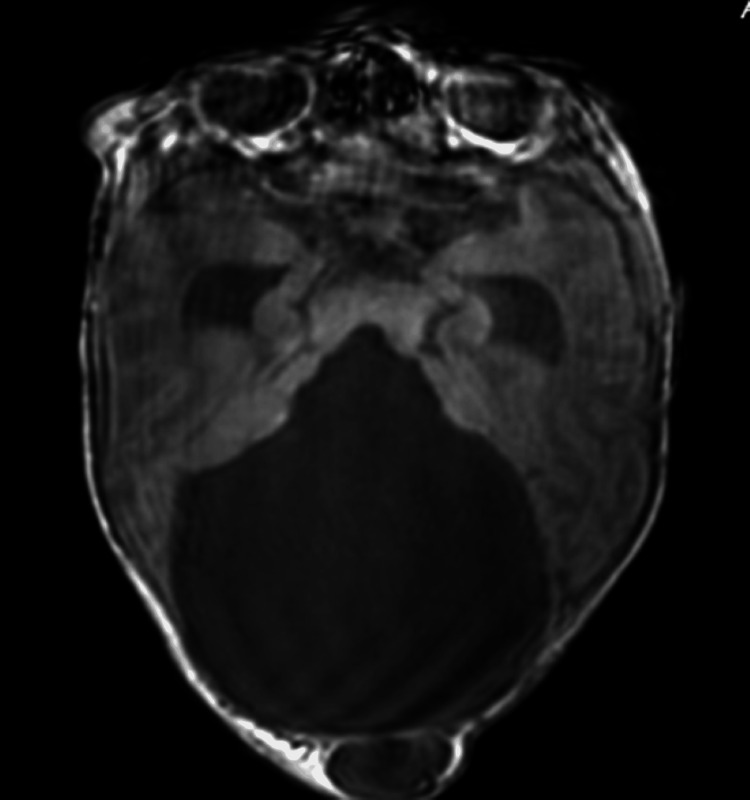
MRI axial image demonstrating the presence of a severe Dandy-Walker malformation and the occipital cephalocele

During his NICU stay, he required respiratory support and experienced recurrent apnea, leading to the suspicion of central apnea. A sleep study confirmed severe central apneas. Postnatally, further diagnostic tests were conducted. A skull X-ray on day one indicated a large soft tissue mass consistent with a cephalocele, while a head ultrasound showed cystic dilatation of the posterior fossa communicating with the fourth ventricle and hypoplasia of the vermis, suggestive of Dandy-Walker malformation. An MRI of the brain performed on day four confirmed a variant of Dandy-Walker Malformation with vermo-cerebellar dysplasia, consistent with Joubert-Plus syndrome, which includes features of both JS (the molar tooth sign) and Dandy-Walker variant (Figure 3). MRI of the spine was normal. 

Whole exome sequencing was undertaken and identified a CPLANE1 mutation, confirming JS.

## Discussion

JS is an uncommon hereditary disorder that was first described in 1969. In JS, neurological manifestations consist of intellectual disability and motor defects. JS shows a defect in the cerebellum and brain stem displaying the “molar tooth” sign, which is practically diagnostic of JS. The disorder can also affect other body systems such as the eyes, the kidneys, and the liver. It can also be associated with other central nervous system abnormalities, such as the Dandy-Walker malformation in the mesencephalon or caudal fourth ventricle. This is known as Joubert-Plus syndrome [6]. 

Dandy-Walker syndrome is a congenital disorder that affects the fourth ventricle and cerebellum. Dandy-Walker variant is a rare condition characterized by cerebellar dysgenesis with or without enlargement of the posterior fossa and variable degrees of cerebellar vermis hypoplasia; the “molar tooth” sign is not present in Dandy-Walker syndrome [6]. Atretic cephalocele is a midline, sub-scalp lesion that contains meninges and may contain neuronal or glial cells covered with skin. Patients with atretic cephaloceles may show normal development or may have significant intellectual disability; this is more likely if the cephalocele is accompanied by intracranial defects such as Walker-Warburg syndrome, ventriculomegaly, or cortical malformations [8]. Recent reports have linked the CPLANE1 gene with ciliopathy, including a mild phenotype of JS and OFDS. Although the association of Joubert-Plus syndrome with cephalocele was described in the literature [5], none of the reports described the association with Dandy-Walker malformation, occipital cephalocele, or Joubert-Plus syndrome caused by the CPLANE1 gene mutation.

Our first patient showed a milder phenotype, as she presented with a suboccipital swelling without signs of early neurological disease such as hypotonia, seizures, feeding difficulty, or abnormal movements; there were no signs of early visual problems. She did show signs of mild motor delay and hypotonia on follow up but has made pleasing developmental progress, in keeping with a mild form of Joubert-Plus syndrome. The heterogeneity of the disorder makes early detection difficult due to the mild symptoms and the difficulties encountered in developmental assessment of neonates and small infants; however, the presence of the cephalocele was a marker for the underlying brain disorder, which was revealed later by the MRI scan and the genetic evaluation. The second patient, who is unrelated to the first patient but carries the same mutation, presented with a severe phenotype, which emphasizes that this disorder has a large spectrum with mild and severe forms. 

The homozygous genetic mutation in the CPLANE1 gene confirmed the diagnosis of JS; most cases in the literature are associated with either OFDS or a mild phenotype of JS, the authors are not aware of a link between Joubert-Plus syndrome with cephalocele and this relatively new gene mutation, especially in the absence of other features of OFDS and skeletal dysplasia which are not present in our two patients. Owing to the high level of consanguinity in our society, JS is not uncommon; we have identified one more local patient with JS due to the CPLANE1 gene, but without the presence of a cephalocele or Dandy-Walker variant or a cephalocele [9].

## Conclusions

Presentation with a subtle occipital swelling should alert to the possibility of an underlying structural brain malformation. An atretic cephalocele is an important marker for underlying brain malformation or genetic syndromes. In such cases, imaging using MRI should be undertaken to confirm the diagnosis. If Joubert-Plus syndrome is being considered, genetic testing should include the CPLANE1 mutation in addition to the other known mutations related to JS. 

## References

[1] González-Gordillo CI, Orozco-Soto LE, Osegueda-Mayen JR, Nava-Tapia A, Martinez-Monreal D (2023). Joubert syndrome: a case report of neonatal presentation and early diagnosis. Bol Med Hosp Infant Mex.

[2] Peraita-Adrados R (2022). Sleep, respiration and nocturnal paroxysmal events in Joubert syndrome: a case report. Nat Sci Sleep.

[3] Mandura RA, Arishi NA (2022). Joubert syndrome presenting with oculomotor apraxia and motor developmental delay: a case report from a neuro-ophthalmology clinic in Saudi Arabia. Cureus.

[4] Shamsudheen MP, Das U, Taduri G, Guditi S, Karthik R, Thakur R (2021). A case of Joubert syndrome with chronic kidney disease. Indian J Nephrol.

[5] Al-Smair A, Younes S, Saadeh A, Kaoukji AR, Jaber O (2022). Joubert-Plus syndrome with an atretic cephalocele: a case report. Radiol Case Rep.

[6] Elhassanien AF, Alghaiaty HA (2013). Joubert syndrome: clinical and radiological characteristics of nine patients. Ann Indian Acad Neurol.

[7] Ahmetgjekaj I, Rahman M, Hyseni F (2021). A case report of Joubert syndrome with renal involvement and seizures in a neonate. Radiol Case Rep.

[8] Kiran RS, Sunitha VC, Kashyap R, Madhan R, Ramesh AS, Nagarajan K (2023). Atretic cephaloceles with different imaging phenotypes - case series with review of literature. J Neurosci Rural Pract.

[9] Bonnard C, Shboul M, Tonekaboni SH (2018). Novel mutations in the ciliopathy-associated gene CPLANE1 (C5orf42) cause OFD syndrome type VI rather than Joubert syndrome. Eur J Med Genet.

